# Society Meetings

**Published:** 1896-07

**Authors:** 


### Society Meetings.

The Medical Society of the County of Chautauqua will hold its annual meeting at Chautauqua, N. Y., Tuesday, July 14, 1896, at 10 o'clock a. m., under the presidency of Dr. E. S. Rich, of Kennedy. A business and scientific program has been arranged for the morning and afternoon sessions by the secretary, Dr. C. A. Ellis, of Sherman. In the evening a banquet will be given at the Hotel Atheneum, which the ladies are specially invited to attend.

THE second Pan-American Medical Congress will convene in the city of Mexico, Tuesday, November 16, 1896, and continue four days.

Dr. William Warren Potter, of Buffalo, vice-president, will receive the titles of papers and furnish information concerning the congress to any who contemplate attending it.

A rare opportunity is afforded in connection with the congress to visit Mexico at a very low rate of fare, and these privileges apply to the families of all members who join the congress. It is important that the names of all such be promptly sent in to Dr. Potter at a reasonably early day.

Prof. Dr. Don Francisco Bastillos, Calle de Tacuba No. 7, Ciudad de Mexico D. F. Republaca Mexicana, has been elected treasurer of the congress, and all members residing in the United States and Canada and others who contemplate attending should forward the registration fee ($5 gold) to him at once and notify Dr. C. A. L. Reed, St. Leger place, Cincinnati.

---

THE Mississippi Valley Medical Association will hold its next meeting at St. Paul, October 20, 21, 22 and 23, 1896, under the presidency of Dr. H. O. Walker, of Detroit. The secretary, Dr. H. W. Loeb, of St. Louis, announces that Dr. H. N. Moyer, of Chicago, will deliver the address on medicine and Dr. Horace H. Grant, of Louisville, that on surgery. Dr. C. A. Wheaton, of St. Paul, is the chairman of the committee of arrangements, and it is announced that all railroads leading thither will offer reduced round-trip rates. The indications are that this will be a very large meeting.

---

THE American Public Health Association will hold its next annual meeting in the building known as Ellicott Square, Buffalo, N. Y., September 15–18, 1896, under the presidency of Dr. Eduardo Licéaga, of the city of Mexico. The executive committee, of which Dr. Ernest Wende is chairman, holds its meetings every Friday between 4 and 5 o'clock, P. M., at the Ellicott Club. It is important that all members of the committee attend promptly, as the meeting will adjourn its sessions positively at the hour named.

---

THE Lake Erie Medical Society will hold its regular quarterly meeting at Angola, Thursday, July 9, 1896. The secretary is Dr. W. W. Jones, Dayton, N. Y.

The American Association of Obstetricians and Gynecologists will hold its ninth annual meeting in the Hotel Jefferson, at Richmond, Va., Tuesday, Wednesday and Thursday, September 22, 23 and 24, 1896, under the presidency of Dr. Joseph Price, of Philadelphia. The meeting promises to be a most interesting one. Mr. Lawson Tait, of Birmingham, Eng., has announced his intention to be present and participate in the proceedings. Other distinguished Fellows and guests will also attend. Dr. George Ben Johnston, of Richmond, is chairman of the committee of arrangements, who will secure hotel accommodations for the Fellows and their guests on application.

---

The Buffalo Microscopical Club held its annual meeting at the library building, Monday evening, June 18, 1896, when the following list of officers was elected for the ensuing year: President, Dr. Herman G. Matzinger ; recording secretary and treasurer, Dr. Jesse Shepard ; corresponding secretary, Dr. Chauncy P. Smith ; advisory council, Dr. Frank J. Thornbury, chairman, Dr. Wm. C. Krauss, Dr. Lee H. Smith. The annual address, entitled A Tribute to Pasteur, was delivered by Dr. Frank J. Thornbury, the retiring president. A meeting of the society will be held on the second Monday in August, to take action regarding the entertainment of the microscopical section of the American Association for the Advancement of Science, to convene in Buffalo, August 22 to 29, 1896.

